# Eosinophilic Granulomatosis With Polyangiitis Diagnosed in an Elderly Female After the Second Dose of mRNA Vaccine Against COVID-19

**DOI:** 10.7759/cureus.21176

**Published:** 2022-01-12

**Authors:** Hanan Ibrahim, Ayad Alkhatib, Alireza Meysami

**Affiliations:** 1 Department of Rheumatology, Henry Ford Health System, Detroit, USA

**Keywords:** covid-19, eosinophilic granulomatosis with polyangiitis, covid-19 mrna vaccine, covid-19 vaccine complication, vaccine, egpa

## Abstract

Eosinophilic granulomatosis with polyangiitis is a type of medium and small-vessel vasculitis that is characterized by asthma, polyneuropathy, peripheral eosinophilia, rhinosinusitis, and other organ involvement, such as the lung and skin. Here, we present an interesting case of eosinophilic granulomatosis with polyangiitis after the mRNA-1273 (Moderna) vaccine against coronavirus disease 2019 (COVID-19). The patient presented with progressive weakness and paresthesia in the upper and lower extremities. She was found to have peripheral eosinophilia and elevated anti-myeloperoxidase antibodies. Nerve and muscle biopsies showed focal vasculitis with infiltration by eosinophils. The patient was started on steroids and a steroid-sparing agent shortly after that and had marked improvement of her symptoms.

## Introduction

Since March 2020, healthcare workers have been fighting against the coronavirus disease 2019 (COVID-19) pandemic. During this battle, tremendous efforts have been put to develop a vaccine to eliminate the spread of COVID-19 variants and protect individuals at high risk for complications. The United States Food and Drug Administration has thus far approved three different vaccines, those of which showed great efficacy and safety rates [1-3].

It has been reported in the literature, however rare, that very few patients developed vasculitis following influenza vaccination [4]. There has also been one case that recently reported eosinophilic granulomatosis with polyangiitis after COVID-19 vaccination [5]. We report here another case of eosinophilic granulomatosis with polyangiitis that was diagnosed after the second dose of COVID-19 mRNA vaccine.

## Case presentation

A 79-year-old Caucasian female with a past medical history significant for adult-onset asthma, gastroesophageal reflux disease, and sleep apnea presented to our hospital’s emergency department for progressive paresthesia of her hands and feet, associated with myalgia and difficulty in ambulation.

The patient came in for progressive worsening of lower and upper extremity paresthesia and pain. Her symptoms started a couple of months before her presentation. She noticed lower back pain radiating down her lower extremities associated with paresthesia and pain in her upper extremities. The patient reported taking supplements including multivitamins, zinc, magnesium, and vitamin C and noticed no change in her symptoms. She started experiencing these symptoms in mid-July 2021 after the second Moderna COVID-19 vaccine dose she received in early July 2021. She initially went to an emergency department, where she was found to have high creatine phosphokinase (424 IU/L; reference range < 178 IU/L) as well as increased absolute eosinophilic count (5.74 K/uL; reference range < 0.70 K/uL) on complete blood count with a mild increase in white blood cells counts (14 K/uL; reference range 3.8-10.6 K/uL). The patient was discharged home with a plan to follow up with her primary care physician. She had worsening pain, weakness, and paresthesia of the upper and lower extremities. She saw her primary care physician and was prescribed gabapentin, which did not help lessen her symptoms. Due to progressive paresthesia and pain, the patient came back to the emergency department a few days after her discharge. During this presentation, the patient reported feeling feverish. She also noted jaw claudication after chewing for some time. She denied headaches, temporal tenderness, or visual changes. She also denied choking episodes, difficulty in breathing, swallowing, or change in her voice, in addition to having any rashes or previous history of joint pain or morning stiffness of the joints prior to her presentation. She denied a history of Raynaud's disease, dry eyes or dry mouth, uveitis, or scleritis. The patient said she had two consecutive miscarriages in her 40s but had three normal pregnancies before that. She denied any blood clots prior to this admission, a history of psoriasis, or inflammatory bowel disease. She also denied a family history of autoimmune conditions.

In terms of past medical history, she was diagnosed with asthma about 20 years ago. Her asthma has been under good control after she was started on inhalers and montelukast. Of note, she denied statin exposure.

She was admitted to the hospital and was noted to have elevated levels of creatine phosphokinase (2093 U/L) and aldolase (29 U/L; reference range 1.2-7.6 U/L). Her complete blood count was significant for the elevation of white blood cells and absolute eosinophilic count to 12.4 K/uL. Due to lower back pain and paresthesia, magnetic resonance imaging of the spine was obtained and showed multiple levels of foraminal stenosis. Doppler ultrasound of her lower extremities showed acute deep venous thrombosis of the mid-calf soleal vein.

Her serology was significant for an anti-myeloperoxidase titer of 103 (reference range titer < 20), rheumatoid factor of 36 IU/mL (reference range < 14 IU/mL), and antinuclear antibody of 1:80 (reference range titer < 1:80). She had a negative myositis panel. Electromyography showed axonal peripheral polyneuropathy that was likely sensorimotor. However, this was not fully discerned due to the extremity edema, with mild myopathic changes in proximal and distal muscles, without membrane irritability. Magnetic resonance imaging showed no findings of myositis.

**Figure 1:**
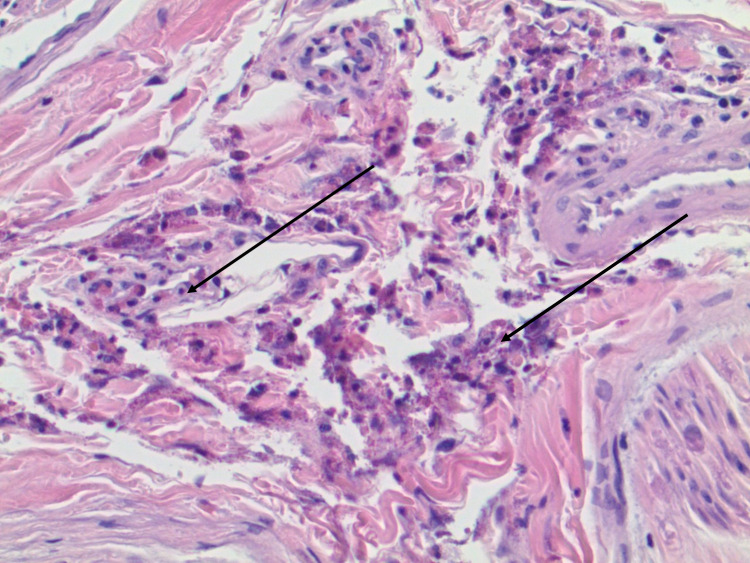
Nerve biopsy showed infiltration by eosinophils.

**Figure 2:**
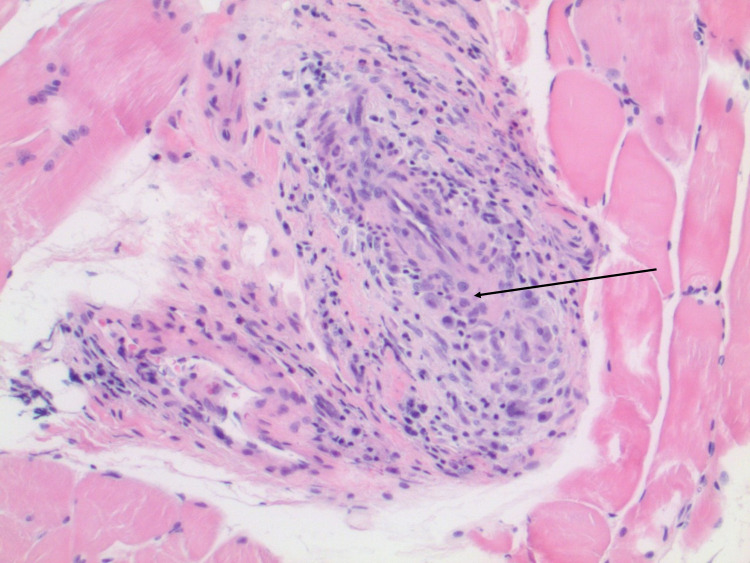
Focal vasculitis involving small perimysial vessels.

Muscle and nerve biopsies were obtained showing focal vasculitis involving small perimysial vessels. However, the muscle was otherwise largely unremarkable. The nerve biopsy showed vasculitis involving a small epineural vessel. Adjacent areas showed infiltration by eosinophils (Figures 1, 2). The biopsy also showed mild pathological features characteristic of axonal neuropathy. She was started on a steroid taper plan with an initial prednisone dose of 60 mg and had improvement of her symptoms. A steroid-sparing agent was offered, and after discussion with her, she was started on azathioprine.

## Discussion

The manifestations of coronavirus disease have been changing and evolving over time. Many researchers are now looking at the effects of the virus and the possibility of it triggering autoimmunity [6]. Several previous reports have linked COVID-19 infection histologically to vasculitis [7]. One Italian observational study found that there was a 30-fold increase in the incidence of Kawasaki-like disease [8]. There are also some reported cases of autoimmune conditions diagnosed after different types of COVID-19 vaccines (e.g., idiopathic thrombocytopenic purpura [9], transverse myelitis [10], Guillain-Barre syndrome [11], and anti-neutrophil cytoplasmic antibody-associated vasculitis [12]), which raises the question if the vaccine might have effects on the immune system similar to the infection itself.

Although the patient already had asthma, she presented with weakness (polyneuropathy), peripheral eosinophilia, and biopsy-proven eosinophilic granulomatosis with polyangiitis a few weeks after her vaccination. There is not enough evidence to suggest causality, whether the vaccine triggered such reaction, revealed an underlying process, or if this was just a coincidence. The patient’s symptoms and deterioration all started after receiving the vaccine, which made us concerned that the vaccine might have had a relation to her presentation. Moreover, as mentioned previously, vasculitis has been reported after influenza vaccination, and the majority of patients that have been identified were females and elderly [4], which is similar to our patient.

Research is still needed to identify the causes behind these cases and to determine if the vaccine triggered an autoimmune kind of process like what was observed in the COVID-19 infected population in the literature [6]. Clinicians must be aware of those cases and must have a high clinical suspicion as early diagnosis and treatment are crucial in such cases to prevent long-term complications.

## Conclusions

Here, we report a case of eosinophilic granulomatosis with polyangiitis after the second dose of the COVID-19 messenger RNA vaccine. Whether it had direct ties to the vaccine or was a coincidence, it emphasizes the importance of considering autoimmune disease after the vaccination in the differential diagnosis. Despite that, vaccination remains crucial during this pandemic fight, and it should not be withheld or restricted.
